# Quantitative analysis of human umbilical vein endothelial cell morphology and tubulogenesis

**DOI:** 10.1111/jmi.13397

**Published:** 2025-02-21

**Authors:** Viviane Mignone, Maria Augusta Arruda, Laura Kilpatrick, Benjamin Moore, Jeanette Woolard, Stephen Hill, Joëlle Goulding

**Affiliations:** ^1^ Division of Physiology, Pharmacology and Neuroscience, School of Life Sciences University of Nottingham Nottingham UK; ^2^ Centre of Membrane Proteins and Receptors (COMPARE) University of Birmingham and University of Nottingham, The Midlands Nottingham Nottingham UK; ^3^ Division of Biomolecular Sciences and Medicinal Chemistry, School of Pharmacy, Biodiscovery Institute University of Nottingham Nottingham UK

**Keywords:** endothelial, HUVEC, ptychography, quantitative phase imaging (QPI), tubulogenesis, VEGF_165_a

## Abstract

Primary human umbilical vein endothelial cells can grow as both a monolayer in culture and also as a capillary‐like network making them an ideal model system in order to study vascular remodelling. Image‐based analysis can allow assessment of cell morphology and motility but is dependent on accurate cell segmentation which requires high‐contrast images not normally achievable without fluorescent markers. Here, ptychography is employed as a label‐free image‐based modality in order to extract quantitative metrics of morphology and tubulogenesis from cultured HUVECs over time in an automated multiwell assay. Phase‐specific parameters of dry mass, optical thickness and sphericity were extracted and assessed alongside other metrics of cell number and shape. Tubulogenesis could be captured dynamically without any imaging artefacts from use of a basement membrane matrix and metrics of tube number, growth and branching exported alongside morphology metrics at early time‐points. Utilising ptychography‐based image analysis, all VEGF_165_a isoforms studied, elicited a concentration‐dependent effect on cell elongation and survival within a HUVEC monolayer. Pharmacologically relevant parameters of potency (EC_50_) and efficacy were derived, exemplifying this label‐free approach for the multiparameter and multiwell quantitative study of vascular remodelling in physiologically relevant cells at 37°C.

## LAY DESCRIPTION

Primary human umbilical vein endothelial cells (HUVECs) are specialized cells that can grow both as a flat sheet (monolayer) and form structures that resemble blood vessels, making them a useful model for studying the changes blood vessels undergo (vascular remodelling). To better understand how these cells behave, researchers often use image‐based techniques to analyse their shape and movement. However, this requires high‐quality, clear images, which are typically achieved by using special fluorescent markers, something that can alter the natural functioning of the cell.

In this study, we used a technique called ptychography, which is a label‐free method for capturing detailed images of the cells. This approach allowed us to study HUVECs in a multiwell setup over time, without the need for fluorescent markers. We measured several important characteristics of the cells, including their mass, thickness, and shape, as well as how they formed blood vessel‐like structures (tubulogenesis).

Additionally, we studied how a specific protein, VEGF165a, and its variants affected the cells, identifying distinct effects on shape, and survival. Using this label‐free approach, we were able to measure key factors like how much of the protein was needed to affect the cells (potency) and how effective it was (efficacy), all at a body temperature of 37°C.

This study highlights the potential of ptychography‐based imaging to analyse complex cell behaviours based on analysing cell shape, growth and morphology alone, offering a more natural and detailed view of vascular remodelling in living cells over time.

## 1 INTRODUCTION

Human umbilical vein endothelial cells (HUVECs) are commonly employed as a cellular model for vascular remodelling,^1^, ^2^, ^3^, ^4^, ^5^ being capable of growing both in a monolayer and also of forming capillary tube‐like structures (tubulogenesis) when seeded on a basement matrix such as collagen or fibrin.^1^, ^5^ Genetic‐, environmental‐ or drug‐induced changes to cell phenotype can be assessed via image‐based analysis,^6^, ^7^ but this depends on accurate cell segmentation (identifying and assigning image pixels into well‐defined objects) and tracking (following object movement through sequential image captures of a time‐lapse series).^8^, ^9^ Accurate segmentation is aided by high‐contrast images^10^ often delivered by employing fluorescent dyes or genetic modifications that encode protein‐tags, however these methods can perturb the endogenous environment and its native responses.^11^, ^12^, ^13^ The alternative is microscopy without these additions, termed label‐free microscopy.^14^ However the most straightforward, affordable and routinely employed modality, bright‐field light microscopy, even when coupled with standard phase^15^ or differential interference contrast (DIC) optics,^16^ produces low contrast images and artefacts such as cell‐halos and shadows which hamper efficient cell segmentation.^17^, ^18^, ^19^ Quantitative phase imaging (QPI) modalities are a family of techniques which measure the phase shift in light as it passes through matter, producing high‐contrast images without aforementioned artefacts.^20^ Ptychography is one such QPI method which retrieves the phase shift computationally through analysis of the intensity shifts captured in a widefield image series.^17^, ^21^ Phase shift occurs when the refractive index (RI; a numerical value depicting the bending of light through a particular medium) changes, for example, as light enters a cell from a liquid environment and equally when passing through the heterogeneous cellular composition of the cell.^22^ Phase shift has been shown to be directly proportional to *dry mass* ^23^, ^24^, ^25^ (biomass without water), and quantification of phase shift also allows the derivation of both the RI and the direct *optical thickness* of the biological matter:^19^, ^23^, ^25^, ^26^

$$\phi =\int \left(\mu_{o}-\mu_{m}\right)\delta t,$$

where ϕ is the measured phase shift, μ the RI of medium (*m*) and object (*o*) and *δt* the change in thickness.

Given the optical thickness and accurate segmentation of a cell, a 3rd metric can be calculated, *sphericity*,^26^ a ratiometric of how closely the cell surface area matches that of a sphere. These phase‐specific measures are not achievable via standard phase contrast or DIC imaging. Aside from this added measurable data, the main advantages of QPI techniques, such as ptychography, are that they are label‐free, utilise low light energy minimising phototoxicity, and can be automated to allow acquisition of large datasets, following multiple acquisition sites over long time periods with minimal user input.^17^, ^20^, ^21^ All such aspects are highly beneficial when using primary cell types.

Vascular remodelling is predominantly regulated by the process of angiogenesis, which is the formation of new blood vessels from a pre‐existing vasculature by the sprouting of endothelial cells towards a pro‐angiogenic stimulus.^1^, ^2^, ^27^ Angiogenesis is strictly controlled by a dynamic balance between pro‐ and anti‐angiogenic factors that ensure the maintenance of the network by regulating endothelial cell survival, proliferation, migration and differentiation.^27^, ^28^ Among the endogenous pro‐angiogenic molecules, vascular endothelial growth factor A (VEGF‐A) is a major regulator of blood vessel formation in health and disease,^29^ signalling through its cognate vascular endothelial growth factor receptor 2 (VEGFR2). Targeting of the VEGF‐A/VEGFR2 signalling axis is a common mechanism of action of therapies used in the treatment of pathologies concerning aberrant blood vessel formation, particularly cancer and macular degeneration.^30^, ^31^, ^32^ The *Vegfa* gene consists of 8 exons and 7 introns, which can be alternatively spliced giving rise to VEGF‐A isoforms that differ in the exons they contain which influences isoform bioavailability, and their abilities to interact with the extracellular matrix and co‐receptors (such as neuropilin).^29^, ^33^, ^34^ VEGF‐A isoforms are termed VEGF_xxx_ according to their number of amino acids. A major site of splicing occurs at the boundaries of exon 8, with proximal splicing resulting in the pro‐angiogenic VEGF_xxx_a isoforms (such as VEGF_165_a, VEGF_121_a, VEGF_145_a and VEGF_189_a) and distal splicing resulting in the weakly angiogenic or anti‐angiogenic VEGF_xxx_b splice variants (eg. VEGF_165_b).^29^ The most recently identified isoform VEGF_165_Ax contains both exons 8a and 8b arising from post‐translational readthrough of the *Vegfa* gene with evidence that it may play both pro‐ and anti‐angiogenic roles.^35^, ^36^, ^37^, ^38^

The purpose of this study was to assess the capability of QPI, namely ptychography, as an imaging modality for the quantitative study of primary HUVECs growing both in a monolayer cell culture and undergoing tubulogenesis. Phase images collected in a label‐free, multiwell automated time‐lapse assay were segmented to allow quantification of cell number and morphology over time and/or subject to an automated network analysis algorithm in order to derive tubulogenic characteristics. We employed this strategy to firstly characterise the morphology of monolayer grown HUVECs overtime assessing whether the effect of growth factor stimulation could be quantified. Secondly we screened VEGF‐A isoforms for their effect on HUVEC monolayer morphology identifying both a concentration‐dependent effect on cell shape and a role for VEGF‐A isoforms in cell survival. Lastly we assessed the native tubulogenic capacity of primary HUVECs and examined the morphological characteristics of single segmented cells within these tube structures.

## 2 MATERIALS AND METHODS

### 2.1 Reagents

All recombinant human VEGF isoforms (#4931) were purchased from R&D Systems (Abingdon, UK). Bovine Serum Albumin (BSA, #A3294) was obtained from Sigma‐Aldrich (Gillingham, UK). Geltrex™ Reduced Growth Factor Basement Membrane Matrix (#A1413201), Medium 200 and Large Vessel Endothelial Supplement (LVES) was purchased from Thermo‐Fisher Scientific (USA).

### 2.2 Cell culture

Human Umbilical Vein Endothelial Cells (HUVECs, passages 4 to 10; #C0035C – single newborn male donor – Thermo‐Fisher Scientific, USA) were grown in Medium 200 supplemented with 2–2.2% LVES (50×) at 37°C, 5% CO_2_. Once they reached 80–90% confluence, they were harvested with trypsin‐EDTA solution (0.25% w/v in versene; Sigma‐Aldrich) counted with a haemocytometer and plated accordingly in Medium 200/2–2.2% LVES (termed complete M200) and left to settle at 37°C, 5% CO_2_ for 24 h.

### 2.3 Cell morphology assay

HUVECs (5 × 10^3^ cells/well) were seeded onto black sided, clear flat bottomed 96‐well plates (#3603, Corning®). Following 24 h of cell growth at 37°C, 5% CO_2_, medium was exchanged for low LVES (0.1%) M200 medium for 24 h. On day 3, the cells were incubated in M200 in the absence or presence of 0.1% LVES and in the absence or presence of VEGF‐A isoforms (100 fM‐3 nM). All M200 media preparations contained 0.1% bovine serum albumen (BSA). The plate was placed in an environmental chamber maintaining the cells within the Livecyte™Cell Imaging System (Phasefocus™, Sheffield, UK) at 37°C, 5% CO_2_ throughout the assay. Image acquisition started after 3 h, to allow the temperature of the plate to settle. The cells were imaged with the Livecyte™Cell Imaging System at 1.5 mm^2^ frame size, every 3 h, for 36 h using a 10× objective (0.25 NA).

### 2.4 Tubulogenesis assay

HUVECs (5 × 10^5 ^cells) were grown in 25 cm^2^ flasks in complete M200. To prepare the 3D matrix, black sided clear, flat bottomed, 96‐well plate (#3603, Corning®) were placed over a cold tray and coated with 50 µL of Geltrex™ (Geltrex™ Reduced Growth Factor Basement Membrane Matrix) and left for 1 h at 37°C to allow polymerisation of the matrix. HUVECs were harvested and plated onto polymerised Geltrex™ (2 × 10^4^ cells/well in M200/2.2% LVES or 1.4 × 10^4^ cells/well in M200/0% LVES in the presence or absence of 1 nM VEGF_165_a). The plate was placed in an environmental chamber maintaining the cells within the Livecyte™Cell Imaging System (Phasefocus™, Sheffield, UK) at 37°C, 5% CO_2_ throughout the assay. Seeded plates were left to acclimatise for 20 min before images were acquired with the Livecyte™Cell Imaging System at 1–1.5 mm^2^ frame size, every hour for 12 h using a 10× objective (0.25 NA).

### 2.5 Image analysis

**FIGURE 1:**
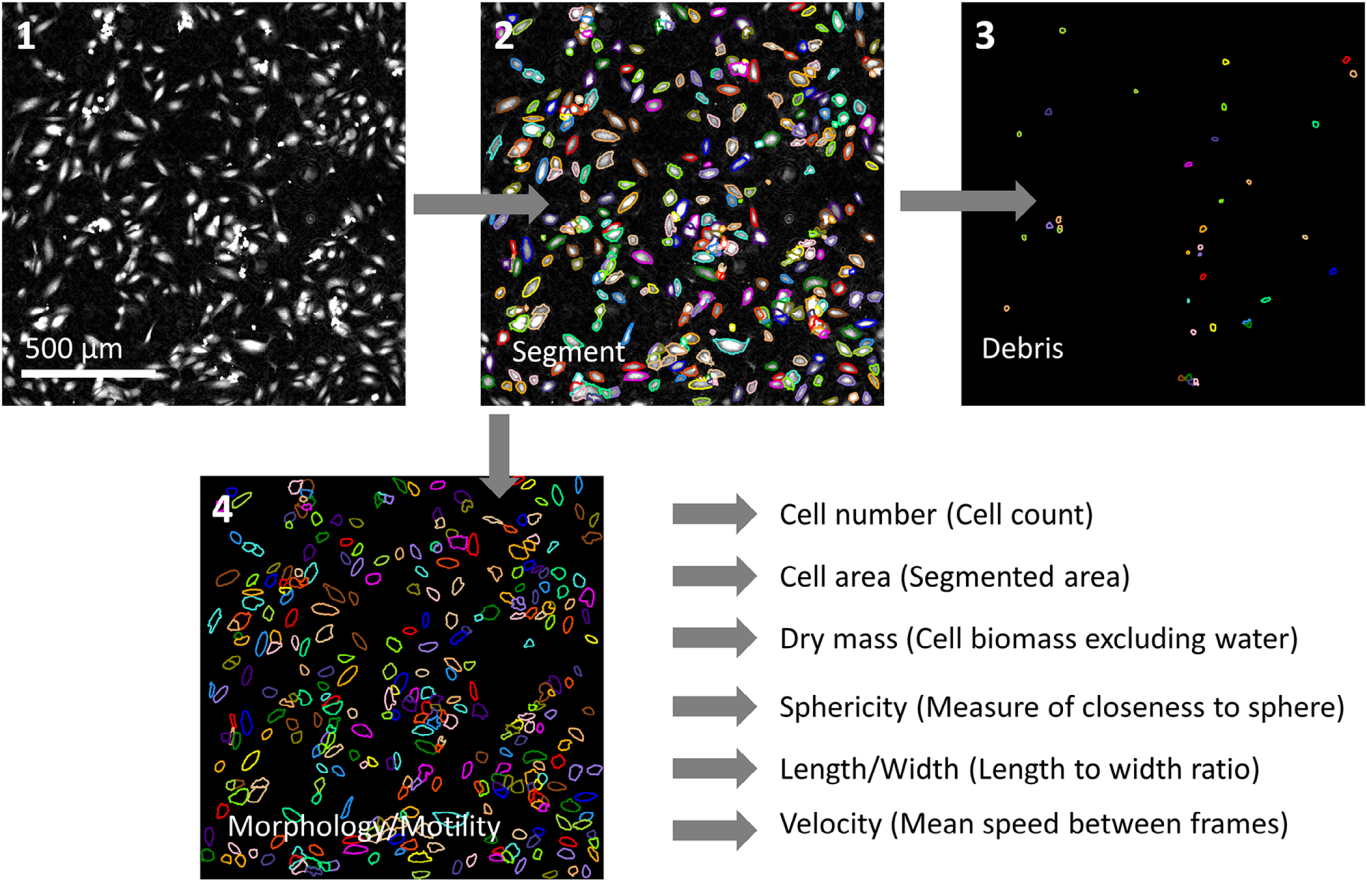
Segmentation of HUVECs using the PhaseFocus™ Cell Analysis Toolbox version 3.8.1. (1) Captured phase images are analysed to (2) identify and segment cells. (3) Regions <400 µm^2^ likely to be debris are removed from the analysis leaving (4) cell data to be collected. Multiple parameters of cell morphology and motility can be extracted.

Acquired ptychography images were reconstructed and analysed within Cell Analysis Toolbox (CAT) software version 3.8.1 (Phasefocus™, Sheffield, UK). For cell morphology assays, images were subject to cell segmentation (Figure 1) with size gates applied to exclude debris by restricting quantification to regions over 400 µm^2^ in area. Initial identification of debris was by eye, with the size gate optimised across multiple images in order to maximise the separation of segmented objects into debris and cell populations. Metrics on morphology were generated from these segmented regions within the CAT software. In this study we have collected Cell Number, Cell Area, Dry Mass, Sphericity, Optical Thickness and Length to Width ratio. Dry mass is the nonaqueous content of the cell given that only biomatter (proteins, nucleic acids, lipids) will cause a phase shift,^20^, ^22^, ^23^, ^24^, ^25^ and can be quantified as protein concentration in picograms.^25^, ^39^, ^40^ Sphericity is a ratiometric measure of how closely the cell shape (cell surface area) matches that of a sphere^26^ and optical thickness (µm, derived from wavelength of light, phase shift and RI) can allow approximation of cell volume if combined with metric data on cell area.^19^, ^24^, ^26^, ^39^

**FIGURE 2:**
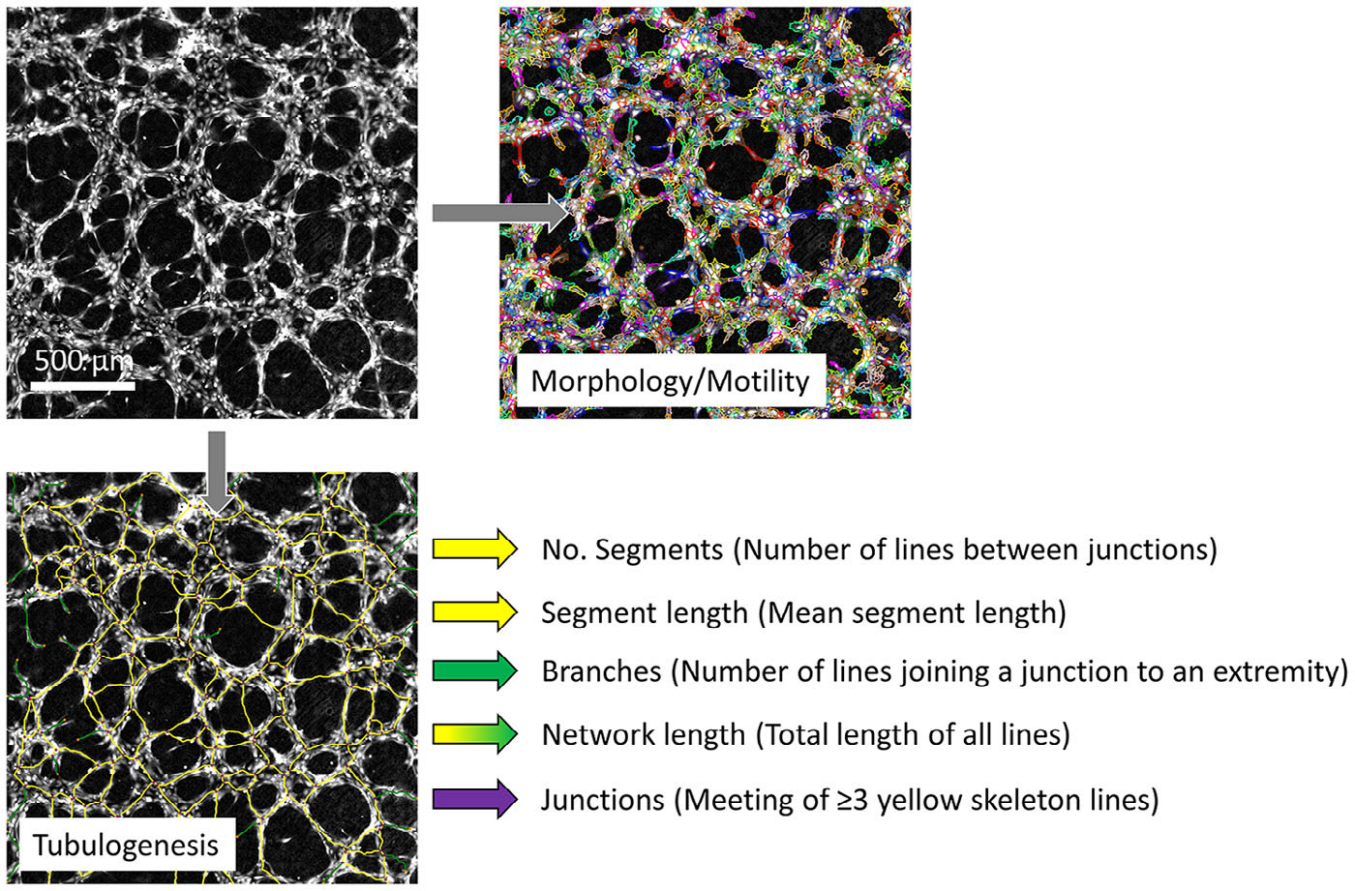
Segmentation and network analysis of HUVECs within a tubulogenesis assay using the PhaseFocus™ Cell Analysis Toolbox version 3.8.1. Morphology, motility and tubulogenesis parameters can be extracted and tracked over time. Morphology/motility image; multicolour overlay depicts segmented cells. Tubulogenesis image; yellow: segment (line between junctions), purple: junction (meeting of ≥3 segments), orange: extremity, green: branch (segment which ends in an extremity).

For tubulogenesis assays, images were subject to an automated network analysis within Cell Analysis Toolbox software version 3.8.1 (Phasefocus™, Sheffield, UK) (Figure 2). The network analysis algorithm was developed by PhaseFocus, utilising a skeletonisation process built on standard (open source) https://OpenCV.org image processing libraries. Extracted metrics were segment number and length, junction and branch number and total network length. Images acquired up until 4 h were also subject to cell segmentation as previously described.

### 2.6 Caspase activity detection

HUVECs (5 × 10^3^ cells/well) were seeded onto black‐sided, clear, flat‐bottomed, 96‐well plates (#655090, Greiner CELLSTAR^®^, UK) and transferred to a low LVES (0.1%) M200 medium for 24 h. On day 3, cells were incubated with VEGF_165_a (100fM‐3 nM/100 µL/well), 1 nM of VEGF_xxx_a isoforms (VEGF_165_b, VEGF_145_a, VEGF_121_a, VEGF_189_a or VEGF‐Ax), 10% LVES or vehicle all diluted in M200/0.1% BSA, in duplicate, for 4–11 replicate experiments. After 48 h at 37°C, 5% CO_2_, the cells were incubated with 2 µM of CellEvent™ Caspase‐3/7 Green Detection Reagent (#C10423, Invitrogen) for 30 min at 37°C. The reagent was aspirated from the wells and the cells were fixed using 3.7% paraformaldehyde in phosphate buffered saline solution (20 min at room temperature) followed by nuclei stained with bisBenzimide H33342 (2 mg/mL; 15 min at room temperature). Images (4 sites/well) were obtained with an ImageXpress Ultra confocal (IXU) high content screening plate reader (Molecular Devices) using a 10× air objective (0.6 NA). The Caspase detection reagent images were obtained using a 488 nm laser and a 525/50 nm emission filter and the bisBenzimide H33342 stained nuclei with a 405 nm laser and 447/60 emission filter. Fluorescence intensity and nuclei count were performed using a modified multiwavelength cell scoring algorithm within the MetaXpress software (MetaXpress 2.0, Molecular Devices). The fluorescence intensity per cell was obtained and the data were normalised to the vehicle treated wells (100%).

### 2.7 Multiplexed morphology and caspase activity assay

HUVECs (5 × 10^3^ cells/well) were seeded onto black‐sided, clear, flat‐bottomed, 96‐well plates (#655090, Greiner CELLSTAR^®^, UK) and the following day transferred to a low LVES (0.1%) M200 medium for 24 h. On day 3, the cells were incubated in M200 in the absence or presence of 0.1% LVES and in the absence or presence of 1 nM VEGF_165_a all diluted in M200/0.1% BSA, in triplicate, for 5 replicate experiments. After 24 h at 37°C, 5% CO_2_, the cells were incubated with 2 µM of CellEvent™ Caspase‐3/7 Green Detection Reagent (#C10423, Invitrogen) for 30 min at 37°C and placed in an environmental chamber maintaining the cells within the Livecyte™Cell Imaging System (Phasefocus™, Sheffield, UK). Single timepoint ptychography and fluorescence (70% CoolLED pE‐300ultra 470–500 nm excitation, 510–540 nm emission, 100 ms exposure) images were acquired with the Livecyte™Cell Imaging System at 1 mm^2^ frame size using a 10× objective (0.25 NA). Images were analysed within CAT as described previously with size gates applied to remove debris (<400 µm^2^) and a threshold included to exclude background fluorescence (<50 fluorescence arbitrary units).

### 2.8 Data analysis and statistical tests

All statistical testing and modelling was done in Prism 10.3.0 (GraphPad Software, San Diego, CA, USA). The following statistical tests were applied; one‐way ANOVA with Dunnett's multiple comparison test for debris comparisons between treatments, two‐way ANOVA with the Geisser–Greenhouse correction, with either Tukey's multiple comparison test for cell morphology comparisons within each time point or Šidák's multiple comparison test for cell morphology comparisons across time points. Mixed‐effects analysis with Dunnett's multiple comparison test for assessing VEGF isoform effect on caspase 3/7 activity. Unpaired *t*‐test for assessing fluorescence intensity to sphericity. Concentration response curves were fit with a three‐parameter nonlinear regression;

$$Y=Bottom+\frac{Top-Bottom}{10^{LogEC_{50}-X}+1},$$

where *Y* is the response (as measured within the assay), *X* is the log of the concentration, and *Top* and *Bottom* refer to the curve plateaus. One‐ or two‐way ANOVAs were applied to derived potency (EC_50_) and efficacy (span) data as stated. All data were expressed as mean ± SEM unless otherwise stated.

## 3 RESULTS

### 3.1 Assessing morphology over time

**FIGURE 3:**
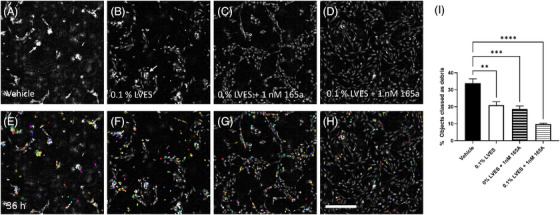
36 h cell morphology. (A–H) Representative ptychography images at 36 h following treatment in the absence or presence of 0.1% LVES and/or 1 nM VEGF_165_a. (A‐B) Arrows depict cell clumping. Panels E‐H show these same images but include multicolour overlays of segmented regions which were <400 µm^2^ and therefore classed as cell debris. These regions were excluded from morphology quantification. (I) Percentage ± SEM of total regions classed as debris from 6–12 separate experiments. Scale bar = 500 µm.

In order to focus on the capability of ptychography to quantify HUVEC morphology over time a monolayer model was applied, where cells are seeded at a low density directly into the multiwell without any additional coating. Individual cells are segmented within each image time point and can be tracked between time points if required. For this study we investigated whether the effects of addition of large vessel endothelial supplement (LVES) and/or the pro‐angiogenic growth factor VEGF_165_a on cell survival, proliferation and morphology could be quantified by ptychography. LVES, as specified by the supplier ThermoFisher, contains a mixture of modulators designed to aid cell growth, migration and angiogenesis: fetal bovine serum, hydrocortisone, human epidermal growth factor, basic fibroblast growth factor, heparin, and ascorbic acid. HUVECs were seeded at a density of 5 × 10^3^ cells/well in the presence or absence of 1 nM VEGF_165_a and/or 0.1% LVES in M200 media, in duplicate wells with 6–12 replicate experiments. Vehicle treatment was the addition of M200 media alone, without LVES. Images were acquired every hour for 36 h and subject to analysis following cell segmentation within the Cell Analysis Toolbox (PhaseFocus™). At 36 h dramatic differences in cell number and health between treatments could be observed visually (Figure 3). Cell number was decreased in wells treated with M200 media alone (vehicle), which lacked both LVES and VEGF_165_a (Figure 3A). Cell clumping and rounding were observed in wells without VEGF_165_a (Figure 3A and B). Debris was identified as segmented regions of area <400 µm^2^ and are shown as multicolour overlays in (Figure 3E–H) with mean ± SEM percentage debris of total segmented regions provided (Figure 3I). Debris was significantly reduced in wells where VEGF_165_a or LVES was present (*p* < 0.0001–0.01, one‐way ANOVA, Figure 3I). In the presence of both 0.1% LVES + 1 nM VEGF_165_a (Figure 3D), cell number appeared to be increased with a smaller percentage of cell debris (9.79 ± 0.53%), as compared to the other treatments.

### 3.2 Quantitative morphology outputs

**FIGURE 4:**
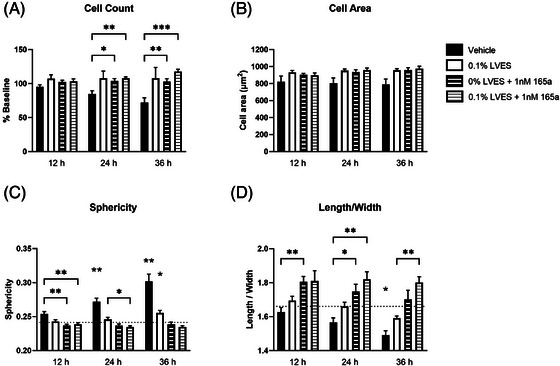
Quantification of cell morphology over time. Morphology‐derived (A) mean cell count, as a percentage of that at *t* = 0 (baseline), (B) mean cell area (µm^2^), (C) mean sphericity and (D) mean length to width ratio as quantified following cell segmentation from ptychography images. Images were acquired at 0, 12, 24 and 36 h following treatment of seeded HUVECs in the presence of vehicle (0% LVES; black bars), 0.1% LVES (white bars), 1 nM VEGF_165_a (thick stripe) or 0.1% LVES + 1 nM VEGF_165_a (thin stripe). Data are mean ± SEM of 12, 6, 7 and 6 separate experiments respectively. Where drawn (C + D) the initial metric value (*t* = 0) is depicted by a dotted line. Significance was tested by two‐way ANOVA and Tukey's multiple comparison test where *p < 0.5, **p < 0.1, ***p < 0.01. Where no line is depicted but significance is illustrated, the selected treatment is significantly different to all other treatments within the select time bin.

Alongside cell number, the morphology metrics of cell area, sphericity and the length to width ratio were quantified at 12, 24 and 36 h (Figure 4). The decrease in cell number following vehicle treatment at 24 h, as normalised to that measured on the initial capture (baseline; *t* = 0), was significant when compared to wells treated with 1 nM VEGF_165_a alone or in combination with 0.1% LVES (*p* < 0.05 & 0.01 respectively; two‐way ANOVA; Figure 4A). Vehicle‐treated cell count was also significantly decreased at 24 h (*p* < 0.01; two‐way ANOVA) compared to that at time = 0 h, no other treatments displayed a significant change in cell number from the initial capture (data not shown). Cell area did not significantly differ between the treatments in any of the time bins (Figure 4B). Cell sphericity, a ratiometric measure of how closely the cell surface area matches the surface area of a sphere of the same volume, can quantify cell health by illustrating the rounding of a cell undergoing the cell death pathway.^41^ Here, sphericity ranged from 0–1, where the complete matching of the cell surface area to that of a hemisphere of the same volume would report a sphericity equal to 1. Mean baseline (time = 0 h) sphericity was 0.24 ± 0.001 (Figure 4C dotted line). Cells without 1 nM VEGF_165_a displayed an increasing ratio over time, steadily become more sphere‐like at each subsequent time point (Figure 4C), at 36 h both vehicle and 0.1% LVES alone treatments show significantly increased sphericity compared to cells stimulated with 1 nM VEGF_165_a (*p* < 0.01 & 0.05 respectively; two‐way ANOVA). The length to width ratio of the cells (Figure 4D) was also affected by the presence of VEGF_165_a, becoming more elongated, compared to initial dimensions (dotted line, 0 h L/W_0_ ∼1.66). Cell elongation following VEGF_165_a treatment was significantly different to that of vehicle after 24 h (*p* < 0.05; two‐way ANOVA). Cells treated with vehicle and those treated with 0.1% LVES and 1 nM VEGF_165_a in combination displayed a significant difference in length to width ratio at 36 h compared to that at the initial capture (time = 0 h, a decrease or increase respectively; data not shown): Vehicle L/W_0_ = 1.67 ± 0.03, L/W_36_ = 1.49 ± 0.03, *p* < 0.0001; 0.1% LVES + 1 nM VEGF_165_a, L/W_0_ = 1.68 ± 0.05, L/W_36_ = 1.80 ± 0.03, *p* < 0.05 (two‐way ANOVA).

### 3.3 VEGF‐_xxx_a isoforms are pro‐survival

**FIGURE 5:**
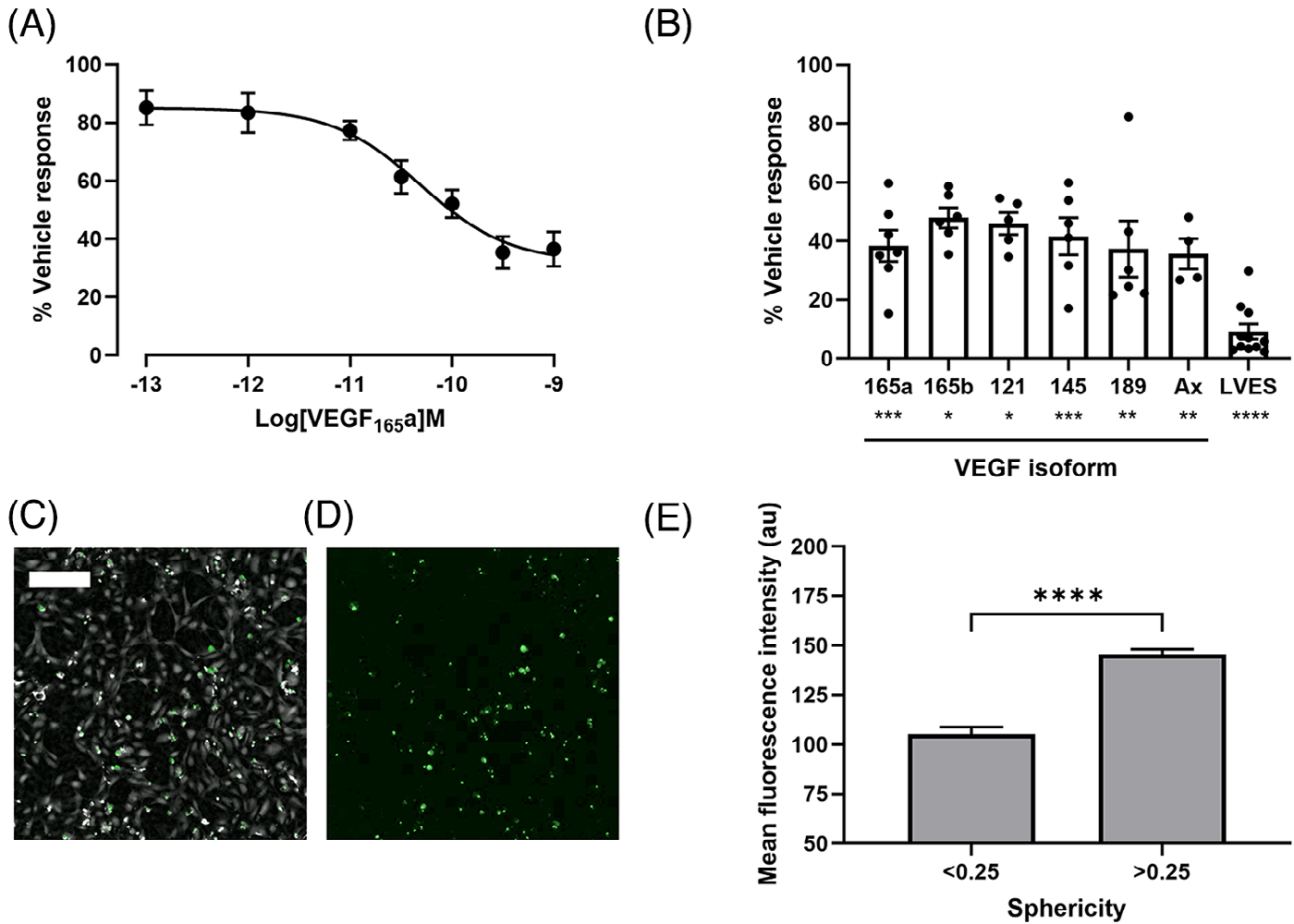
VEGF_xxx_a effect on caspase activity. (A) VEGF_165_a concentration response curve of caspase activity after 48 h treatment. Data are normalised to the caspase activity measured following treatment with vehicle (100%). Data are mean ± SEM of 6 separate experiments. (B) Caspase activity measured at 48 h following treatment with 1 nM of stated VEGF_xxx_a isoform in vehicle or after treatment with 10% LVES. Data are normalised to the caspase activity measured following treatment with vehicle (100%). Data are mean ± SEM of 3–11 separate experiments; each experiment is represented as a single data point. Significance was tested with Dunnett's mixed comparison test where *p < 0.05, **p < 0.01, ***p < 0.001 or ****p < 0.0001. (C) Representative merged ptychography and fluorescence (caspase activity, green) image and (D) fluorescence only image. Scale bar = 200 µm. (E) Mean fluorescence intensity ± SEM (arbitrary units) of pooled single cell data from 5 separate experiments, grouped as less than or greater than 0.25 sphericity. Significance was tested with an unpaired *t*‐test where ****p < 0.0001.

The dramatic effect on cell sphericity in the absence of VEGF_165_a, (Figure 4C) supports the previously reported role for VEGF_165_a in endothelial cell survival.^42^, ^43^, ^44^, ^45^ To follow up our morphology findings, the caspase 3/7 activity of HUVECs treated with VEGF_165_a after 48 h was assessed using CellEvent™ Caspase‐3/7 Green Detection Reagent. Caspase‐3 and ‐7 are known as *effector* caspases, which go on to cleave substrates, bringing about programmed cell death.^46^ In addition to VEGF_165_a we tested the caspase 3/7 activity of 5 other VEGF_xxx_a isoforms to screen for a conserved pro‐survival function. HUVECs were seeded at a density of 5×10^3^ cells/well in the presence or absence of VEGF_xxx_a isoforms at varying concentrations in minimal media (0% LVES). VEGF_xxx_a caspase activity was normalised to that seen from cells incubated in vehicle alone (100%). VEGF_165_a elicited a concentration‐dependent effect on caspase activity, with a potency, pEC_50_, of 10.37 ± 0.21 and a maximal repression to 36.6 ± 5.93% (Figure 5A). At their highest tested concentration (1 nM) all VEGF_xxx_a isoforms were able to repress caspase 3/7 activity to a comparable extent, with 10% LVES (representing a positive control) repressing activity to 9.13 ± 2.57% (Figure 5B). Repression of caspase 3/7 activity was significant for all isoforms when compared to that observed following treatment with vehicle alone (Dunnett's multiple comparison test, *p* < 0.05–0.0001; Figure 5B). To further confirm a relationship between sphericity and cell death, multiplexed ptychography and fluorescence images were captured in a separate experiment following 24 h treatment in the presence or absence of VEGF_165_a and/or LVES and a subsequent 30 min incubation with CellEvent™ Caspase‐3/7 Green Detection Reagent. Both diffuse regions and intense fluorescence spots, depicting regions of varying caspase 3/7 activity, could be observed within the cultured HUVECs (Figure 5C and D). Segmented objects were again subject to size gating to remove debris and a threshold applied to exclude background fluorescence. This multiplexed assay meant that morphology metrics for each segmented cell could be paired with fluorescence data. From pooled single‐cell data, collected over 5 separate experiments, cells with a sphericity over 0.25 displayed a significantly increased mean fluorescence intensity, depicting higher caspase‐3/7 activity, as compared to cells with a sphericity less than 0.25 (unpaired *t*‐test, *p* < 0.0001; Figure 5E). The boundary of 0.25 was chosen given that both baseline sphericity (0 h) and growth factor treated cells (24 h) displayed a sphericity <0.25 (Figure 4C).

### 3.4 VEGF_xxx_a isoforms display a concentration‐dependent effect on cell elongation

**FIGURE 6:**
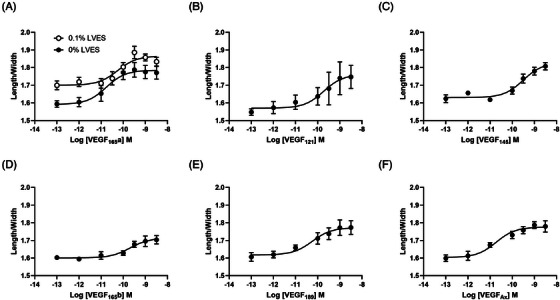
VEGF_xxx_a concentration‐dependent effect on cell shape. Concentration response curves of mean length to width ratios derived from ptychography images at 12 h following treatment with (A) VEGF_165_a in 0.1% LVES (open circles) or vehicle (0% LVES; closed circles), (B) VEGF_121_a in vehicle, (C) VEGF_145_a in vehicle, (D) VEGF_165_b in vehicle, (E) VEGF_189_a in vehicle or (F) VEGF‐Ax in vehicle. Data are mean ± SEM of 3–7 separate experiments (see Table 1).

We then assessed whether the effect of VEGF_165_a on cell elongation observed in Figure 4D, in terms of length to width ratio, was conserved between isoforms and whether it was dependent on stimulus concentration. The effect of 6 different VEGF_xxx_a isoforms on cell length to width ratio was examined covering a concentration range of 100 fM – 3 nM over 36 h. HUVECs were seeded at a density of 5 × 10^3^ cells/well in the presence or absence of varying VEGF_xxx_a concentrations, but in the absence of LVES. VEGF_165_a was also examined in the presence and absence of 0.1% LVES. Treatments were carried out in duplicate wells with 3–7 replicate experiments as detailed in Table 1. Images were acquired every hour for 36 h and subject to analysis following cell segmentation within Cell Analysis Toolbox (PhaseFocus™). At 12 h (Figure 4D), 1 nM VEGF_165_a had already elicited an effect on cell elongation and this was confirmed as a concentration‐dependent effect in both the presence and absence of 0.1% LVES (Figure 6A). Wells treated with VEGF_165_a in the presence of 0.1% LVES displayed an elevated minimal and maximum length to width ratio while potency and span (max‐min effect) were not significantly different (Figure 6A, Table 1). Similarly all other tested VEGF_xxx_a isoforms elicited an equivalent concentration‐dependent effect on length to width ratio at 12 h (Figure 6, Table 1). At later time points (24 h and 36 h) this concentration‐dependent effect on length to width ratio was maintained however in the absence of LVES, measurements were depressed in amplitude (Figure S1c–h, Table 2). In contrast, for cells treated with VEGF_165_a + 0.1% LVES, maximal length to width ratio was maintained at both 24 and 36 h (Figure S1a). It is likely that the time‐dependent reduction in length to width ratio may relate to the opposing force of deteriorating cell health at the extended time points of 24 and 36 h, illustrated by increasing sphericity. The converse concentration‐dependent decrease in sphericity could be observed with increasing VEGF_165_a concentrations (Figure S1b, Table 2) which also displayed a converse time‐effect on maximal response. These responses were severely dampened in the presence of 0.1% LVES.

**TABLE 1:** Potency (pEC_50_) and efficacy (span) of VEGF_xxx_a isoforms with respect to effect on the mean length to width cell ratio at 12 h (Figure 6).

Isoform	pEC_50_	SEM	*n*	Span	SEM
VEGF_165_a + 0.1% LVES	10.34	0.09	7	0.17	0.02
VEGF_165_a + 0% LVES	10.78	0.24	5	0.20	0.02
VEGF_121_a	9.44	0.32	3	0.21	0.05
VEGF_145_a	9.55	0.20	5	0.21	0.02
VEGF_165b_	9.98	0.48	4	0.12	0.03
VEGF_189_a	10.24	0.27	5	0.16	0.03
VEGF‐Ax	11.82	0.39	6	0.20	0.03

*Note*: There was no significant difference between isoform pEC_50’_s nor span as tested with one‐way ANOVA.

### 3.5 Assessment of tubulogenesis through QPI

An important aspect of using HUVECs as a model of angiogenesis is their ability to spontaneously form capillary‐like tubes emulating their tubulogenic function. This behaviour is dependent on a basement matrix; in our case, we have seeded cells onto Geltrex™. We next assessed whether ptychography was able to capture this tubulogenic nature, without imaging artefacts due to a basement matrix, and then to deliver metrics that could allow quantitative assessment of this procedure over time.

**FIGURE 7:**
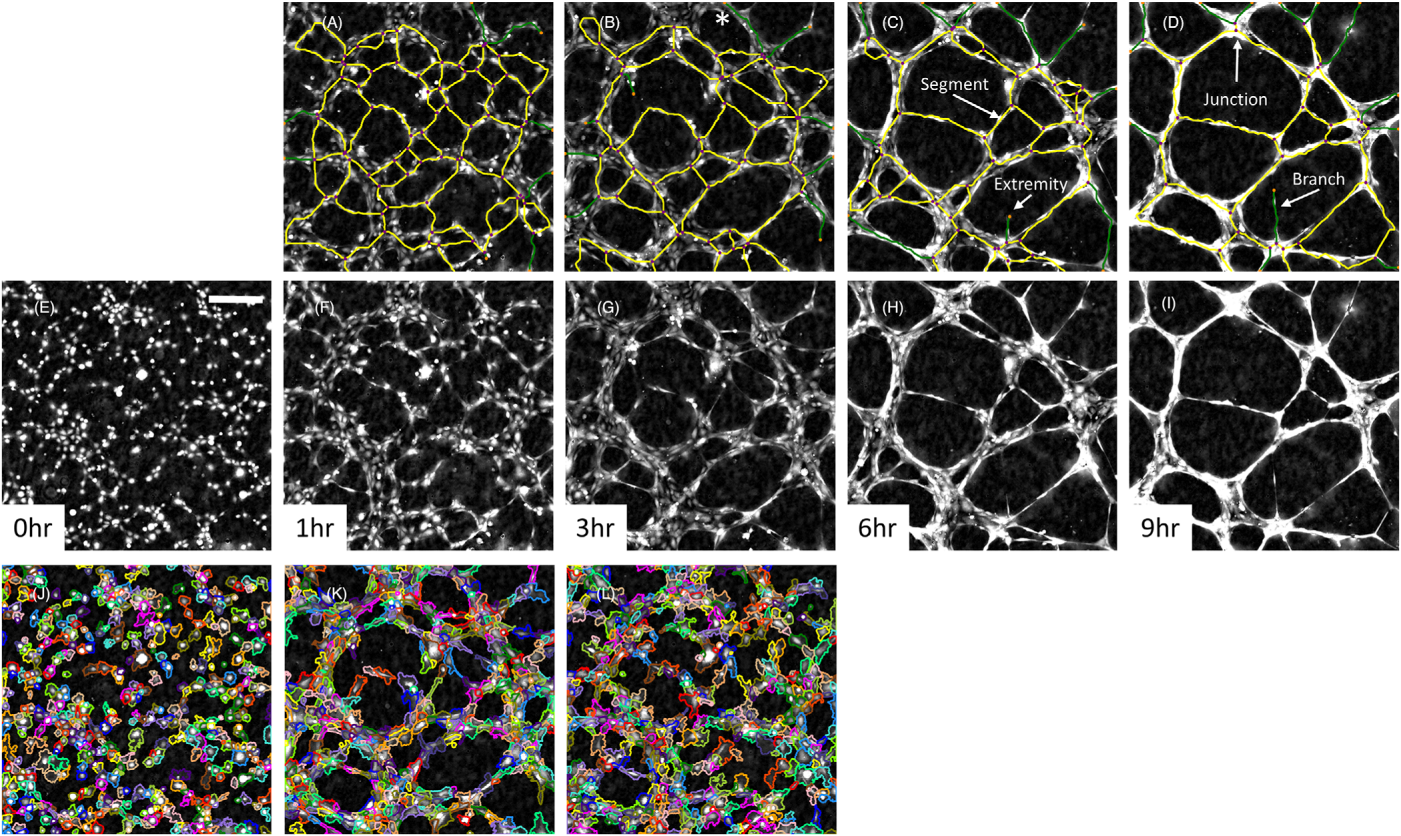
Spontaneous tube formation. (A–L) Representative ptychography images over time of HUVEC cells seeded on Geltrex™ coated 96‐well plate in M200 media with 2.2% LVES. Scale bar = 200 µm. (A–D) Overlay depicts automated network analysis with segments (yellow), branches (green), junctions (purple) and extremities (orange). Asterisk (*) illustrates potential mislabel of segment as a branch at image boundary. (J–L) Overlay depicts cell segmentation.

**FIGURE 8:**
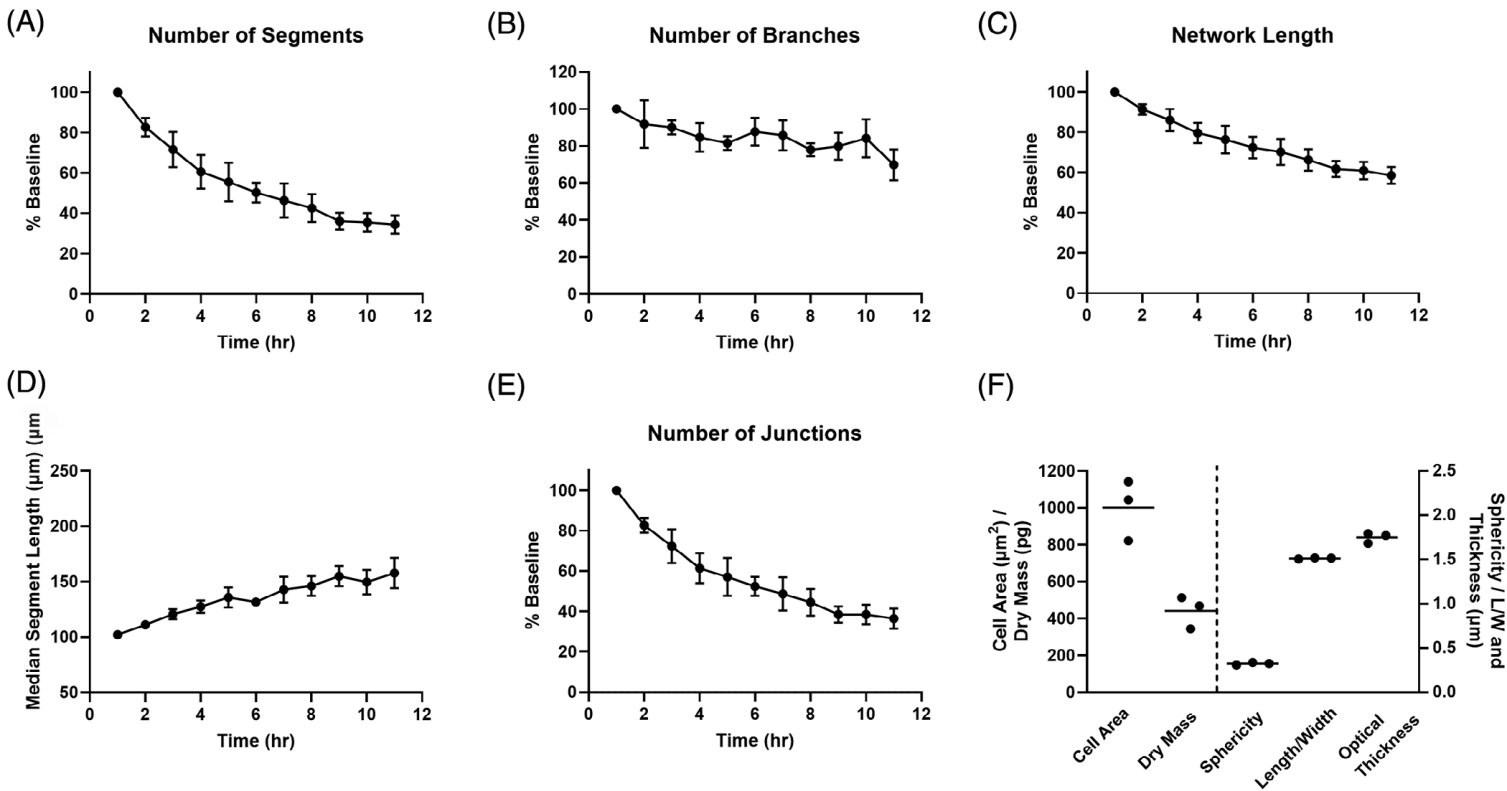
Quantifying tubulogenesis in the presence of 2.2% LVES. Tubulogenesis parameters, as a percentage of that seen at 1 h where appropriate, to depict number of segments (A), branches (B), network length (C), median segment length (D) and number of junctions (E). Data are mean ± SEM of 3 separate experiments, where 5 images were analysed per experiment. (F) Raw morphology parameters derived from cell segmentation at 3 h. Data are mean (line) and raw median value from 3 separate experiments.

Primary HUVECs were seeded at 2 × 10^4^ cells/well on polymerised Geltrex™ in M200 media supplemented with 2.2% LVES and imaged over 11 h. The experiment was repeated 3 times with each experiment consisting of 5 replicate wells. After 1 h, the beginning of networks could already be observed without any imaging artefacts that would otherwise hinder automated analysis (Figure 7F). As time progressed the network became more defined, quickly being reorganised from a monolayer to a network of tubes whose thickening could be seen through an increase in contrast/opacity after 6 h (Figure 7E–I, Video S1). Tubulogenesis was assessed by applying the automated network analysis within the Cell Analysis Toolbox (PhaseFocus™) and deriving network‐based metrics, such as segment length and number, branch and junction number (Figure 7A–D and Figure 8A–E). Up until 3–4 h, separate cells could still be segmented and morphologic parameters extracted, however segmentation should be assessed by eye to assess degree of segmentation errors, for example, multiple cells being segmented as one (Figure 7J–L and Figure 8F). After this time point separate cells could no longer be faithfully segmented.

As can be seen in both time‐lapse images (Figure 7) and plotted parameters (Figure 8A and D), the number of tube segments steeply dropped as time progressed alongside the median segment length increasing. Coupled with the junction number and total network length decreasing (Figure 8C and E) these parameters illustrate coalescing of cells and tubes into fewer but thicker segments. Branches are defined as segments which end in an extremity and could model reaching/searching protrusions.^47^ However data should be treated with caution as branches are often observed at image boundaries (Figure 7B asterisk). Sequential images should be examined to determine true branch versus segment designation. At 3 h, cells could still be segmented and morphology parameters extracted, segmentation errors were minimal and not concentrated in a particular treatment (Figure 7L and 8F). Cell area and dry mass showed a little variability however experimental variation was maintained, whereby the experiment where cells had the largest median cell area was also the experiment with the biggest median dry mass. Sphericity, length to width ratio and optical thickness of HUVECs were closely maintained between experiments.

### 3.6 Exogenous VEGF_165_a does not affect tube formation

**FIGURE 9:**
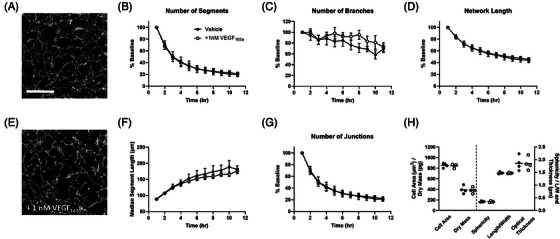
HUVEC tubulogenesis following VEGF_165_a stimulation. Representative ptychography image captured at 3 h illustrating HUVECs in minimal M200 media and in the absence (A) or presence (E) of 1 nM VEGF_165_a. Scale bar = 500 µm. Tubulogenesis parameters, as a percentage of that seen at 1 h where appropriate, to depict number of segments (B), branches (C), network length (D), median segment length (F) and number of junctions (G) in the presence (open circles) or absence (closed circles) of 1 nM VEGF_165_a. Data are mean±SEM of 4 separate experiments. (H) Raw morphology parameters derived from cell segmentation at 3 h. Data are mean (line) and raw median value from 4 separate experiments.

Given that HUVEC tubulogenesis could be modelled and quantified via ptychography we then tested whether HUVEC tube generation was modulated by exogenous VEGF_165_a. Primary HUVECs were seeded at 1.4 × 10^4^ cells/well on polymerised Geltrex™ in minimal M200 media (without LVES supplementation) and imaged over 11 h. The experiment was repeated 4 times with each experiment consisting of duplicate wells. LVES itself contains growth factors, human epidermal growth factor and basic fibroblast growth factor and was therefore omitted to prevent potential masking of VEGF_165_a mediated effects. In the absence of VEGF_165_a and LVES, HUVECs seeded on polymerised Geltrex™ were able to spontaneously undergo tubulogenesis (Figure 9A), as has been noted previously.^48^, ^49^ As seen earlier following LVES supplementation, cells gradually coalesced into tubes with median segment length increasing while the number of junctions and segments decreased (Figure 9B, F and G). No significant difference was observed in the generation of tubes in the absence of LVES compared to that observed with 2.2% LVES (data not shown; two‐way ANOVA). Nor did the addition of exogenous VEGF_165_a (1 nM) modulate HUVEC tubulogenesis or indeed cellular morphology; metrics extracted at the 3 h time point were closely aligned between conditions (Figure 9E and H).

**TABLE 2:** Potency (pEC_50_) of VEGF_xxx_a isoforms with respect to effect on the mean length to width cell ratio or sphericity at 12, 24 and 36 h (Figure S1).

Length/width							
Isoform	pEC_50_ 12 h	SEM	pEC_50_ 24 h	SEM	pEC_50_ 36 h	SEM	*n*
VEGF_165_a + 0.1% LVES	10.34	0.09	10.21	0.22	10.11	0.21	7
VEGF_165_a + 0% LVES	10.78	0.24	10.39	0.28	10.49	0.29	5
VEGF_121_a	9.44	0.32	9.70	0.39	9.78	0.23	3
VEGF_145_a	9.55	0.20	9.39	0.21	9.30**	0.22	5
VEGF_165b_	9.98	0.48	9.53	0.41	10.07**	0.30	4
VEGF_189_a	10.24	0.27	9.99	0.30	9.95	0.19	5
VEGF‐Ax	11.82	0.39	10.17	0.36	10.17	0.27	6

Sphericity							
Isoform	**pEC_50_** **12 h**	**SEM**	**pEC_50_** **24 h**	**SEM**	**pEC_50_** **36 h**	**SEM**	*n*
VEGF_165_a + 0.1% LVES	10.29	0.43	10.21	0.19	10.14	0.14	7
VEGF_165_a + 0% LVES	11.10	0.24	10.78	0.21	10.87	0.20	5

*Note*: Any significance in potency as determined by length to width ratio, between isoforms within time bins was tested by one‐way ANOVA with Tukey's multiple comparison test. At 36 h VEGF_145_a and VEGF_165_b were significantly different, **p < 0.01. There was no significant difference in potency as determined by sphericity for cells treated with VEGF_165_a in the absence or presence of 0.1% LVES at any time‐point as tested by two‐way ANOVA.

## 4 DISCUSSION

In this study, we demonstrate that quantitative phase imaging, namely, ptychography, can be a valuable tool to facilitate the use of HUVECs as a model of vascular remodelling. These cells will grow in a monolayer but can also spontaneously form tube‐like structures, mimicking vasculogenesis and angiogenesis, when seeded on an appropriate basement membrane.^1^, ^5^, ^48^, ^49^ HUVECs, however, are primary cells obtained from donor umbilical cord, whose phenotype will alter as they are passaged and age.^1^, ^50^, ^51^, ^52^ Not only therefore is an imaging tool required that permits complex image retrieval in order to maximise data output from a valuable primary cell source but also one that does not perturb the natural function of the cells in question. Ptychography‐based imaging is able to deliver on both these elements.^7^, ^17^, ^20^, ^21^

Ptychography is a label‐free imaging technique utilising low intensity illumination for acquisition, minimising phototoxicity and foregoing the need for genetic modification or exogenous dye addition in order to identify cells.^17^, ^20^, ^40^ We demonstrate in this study that HUVECs can be segmented and their native morphology quantified via ptychography‐based image analysis. In addition tubulogenic metrics can be extracted that describe the formation of tubes dynamically without any imaging artefacts from use of a basement membrane coating. We have previously used this technique to characterise CRISPR/Cas9 gene‐edited immortalised HUVECs (TERT2 HUVEC) to ensure the retention of this spontaneous tube formation but only as an end‐point measure.^51^ In this study, we demonstrated that early passage (P4–10) HUVECs will spontaneously form tube‐like structures when seeded on the reduced growth factor basement membrane matrix, Geltrex™, in the absence of additional growth factors or supplements. Utilising an exogenous dose of VEGF_165_a (1 nM), which is in keeping with the known affinity of VEGF_165_a for the VEGFR2,^29^ we did not identify any VEGF_165_a‐ nor any LVES‐driven modulation of tube formation and growth. It has been demonstrated that a 20‐fold higher dose of VEGF_165_a is able to increase total network length and branch formation.^48^ Guo et al. employed a different reduced growth factor basement membrane matrix, Matrigel, and assessed tube formation from a 1 mm^2^ frame size at a single time point (4–5 h). Cultured HUVECs have been shown to secrete endogenous VEGF‐A, alongside other growth factors, which have been implicated in the modulation of cell proliferation and migration.^53^, ^54^, ^55^, ^56^ This secretion of VEGF‐A has been shown to be upregulated following environment stress factors such as hypoxia^56^ and low seeding density.^54^ It is possible, therefore, that the endogenous secretion of VEGF_165_a within our study is sufficient to maximally activate tube formation by HUVECs when seeded on a basement matrix.

Ptychography provides phase‐specific output metrics of morphology^22^, ^23^, ^24^, ^25^, ^26^ (dry mass, optical thickness and sphericity), alongside high contrast images which are essential for high‐accuracy cell segmentation and standard morphology outputs of parameters such as cell area and length to width ratio. In addition cell segmentation and an appropriate time‐lapse regime permits cell tracking which can be applied to follow cell migration. It is possible therefore to infer cell fate via specific morphological parameters,^8^ rather than by expression or activation of signalling molecules or reporter genes. One clear example in this work is sphericity as a measure of cell death. Within this study increased cell death, apparent visually as reduced cell number, cell clumping and increased debris, could be quantified by the increasing sphericity of cells over time. In a multiplexed assay increased caspase 3/7 activity, a marker of programmed cell death,^46^ was observed in the cell population with the greater sphericity. Cell survival roles can hence be identified, for example VEGF_165_a displayed a concentration‐dependent effect, with addition of higher concentrations leading to reduced sphericity. This role for VEGF_165_a in promoting cell survival was supported with the observation of a concentration‐dependent decrease in caspase 3/7 activity. The VEGF_165_a cell survival role has been observed previously through its effect on cell death marker and effector levels and/or activity in endothelial cells^42^, ^43^, ^44^, ^45^ however not from its direct morphological effect and nor in a real‐time manner. We were also able to identify a concentration‐dependent effect on cell elongation for all the screened VEGF_xxx_a isoforms. Increased VEGF_xxx_a doses led to a greater length to width ratio. This effect on cell shape has been already described with VEGF levels implicated in the mediation of transcription factor‐ and signalling molecule‐driven cell elongation.^57^ Indeed it has been suggested that endothelial cell elongation is required for vascular like morphology and remodelling^2^ potentially to facilitate the protrusive nature of endothelial cells during vascularisation.^47^ Concerning cell shape, however, the particular assay selected was important, as in this study the increase in length to width ratio was only observed in the monolayer morphology assays and not the tubulogenesis assays. At the 3 h time point in the tubulogenesis assays, cells could still be segmented, however the length to width ratio in all conditions (minimal media, LVES supplemented media and VEGF_165_a supplemented media) was maintained at ∼1.5 which contrasted with the elongated cells observed after treatment with 1 nM VEGF_165_a in a cell monolayer (0% LVES 1.79; 0.1% LVES 1.87). This environment‐dependent morphology has been previously reported where HUVEC morphology, biochemistry and growth differ when grown on an extracellular matrix (Matrigel) or on gelatin alone.^58^

This study was carried out in a multiwell plate with experimental conditions performed in duplicate or triplicate. While not a high‐throughput approach this automated format, with minimal user interaction is ideal for derivation of a wide range of pharmacologically relevant outputs. Once an effect on cell shape (sphericity and length to width ratio) following growth factor stimulation was identified it was possible to design an experiment, utilising multiple drugs and doses, in which the drug potency (pEC_50_) and efficacy (span) could be quantified from images. Indeed the extracted pEC_50_ values for sphericity, suggesting progression along the cell death pathway (VEGF_165_a 36 h 0% LVES; 10.87 ± 0.20), was comparable to that extracted from the plate‐based caspase 3/7 activity assay (10.37 ± 0.21). The VEGF_165_a‐driven effect on cell elongation also displayed a similar potency value (10.78 ± 0.24), all of which are presenting within a physiologically relevant concentration of VEGF_165_a (∼50 pM).

The derivation of phase‐specific quantifiable metrics is not limited to ptychography, other QPI techniques, namely interferometry^20^, ^59^, ^60^, ^61^ and the related methodologies of digital holography^60^, ^62^ and wavefront sensing,^61^, ^63^ have also demonstrated their use with biological samples. While ptychography retrieves the phase‐shift computationally from the intensity changes in a widefield image series, these other techniques employ extra elements within the beam‐path either to create dual light rays (one reference and one passing through the sample) or interference patterns, for example, through insertion of a grid or lens array.^39^, ^60^, ^61^ Of late the most recent adaptions have moved towards establishing the phase‐retrieval of 3D structures, for example TBFI (tomographic bright field imaging),^64^ which, like ptychography, utilises a standard imaging set‐up and compares intensity fluctuations, but incorporates a very narrow aperture in order to produce a unidirectional plane wave that can report on the axial deviations. Slight differences in spatial and temporal resolution alongside signal to noise can be found between these techniques, as reviewed comprehensively by Nguyen *et al.*;^20^ however, ptychography may be thought of as the most accessible, not requiring extra or specialised optical elements and indeed commercially available in a multiwell automated format and open to multiplexing as we have shown here. Ptychography has allowed us to confirm both the functional effects on HUVECs following stimulation by a well described growth factor, VEGF‐A, but also derive valuable pharmacological parameters (potency, efficacy) of such effects. Coupled with image‐based cell phenotype deep‐learning^8^ this tool has the potential to enhance fundamental and industry level research into endothelial cell function.

## 5 GLOSSARY

**Angiogenesis**: the process of the formation of new blood vessels from a pre‐ existing vasculature.

**Efficacy**: the maximal effect of a stimulus.

**Phase shift**: the change in the phase of the waveform of light as it passes through matter.

**Potency (EC_50_)**: a measure of the concentration of drug required to elicit half the maximal drug effect.

**Ptychography**: imaging modality which retrieves the phase shift of light computationally through analysis of the intensity shifts captured in a widefield image series.

**Quantitative Phase imaging**: collective term for imaging modalities which retrieve phase shift of light as it passes through matter.

**Refractive Index (RI)**: a numerical value depicting the bending of light through a particular medium.

**Tubulogenesis**: the process of forming tube‐like structures.

## Supporting information



Figure S1. Concentration response curves of mean length to width ratio (a, c–h) or mean sphericity (b) derived from ptychography images at 12 h (black), 24 h (red) and 36 h (green) following treatment with (a) VEGF_165_a in 0.1% LVES (open circles), (b) VEGF_165_a in 0.1% LVES (open circles) or vehicle (0% LVES; closed circles), (c) VEGF_165_a in vehicle (0% LVES; closed circles), (d) VEGF_121_a in vehicle, (e) VEGF_145_a in vehicle, (f) VEGF_165_b in vehicle, (g) VEGF_189_a in vehicle or (h) VEGF_165_Ax in vehicle. Data are mean ± SEM of 3–7 separate experiments (see Table 2).

Supporting information
