# Supplementary figures and images for: Quantitative analysis of human umbilical vein endothelial cell morphology and tubulogenesis
Source: J Microsc. 2025 Feb 21;300(2):234–49. doi: 10.1111/jmi.13397 (PMC12523982; doi:10.1111/jmi.13397)

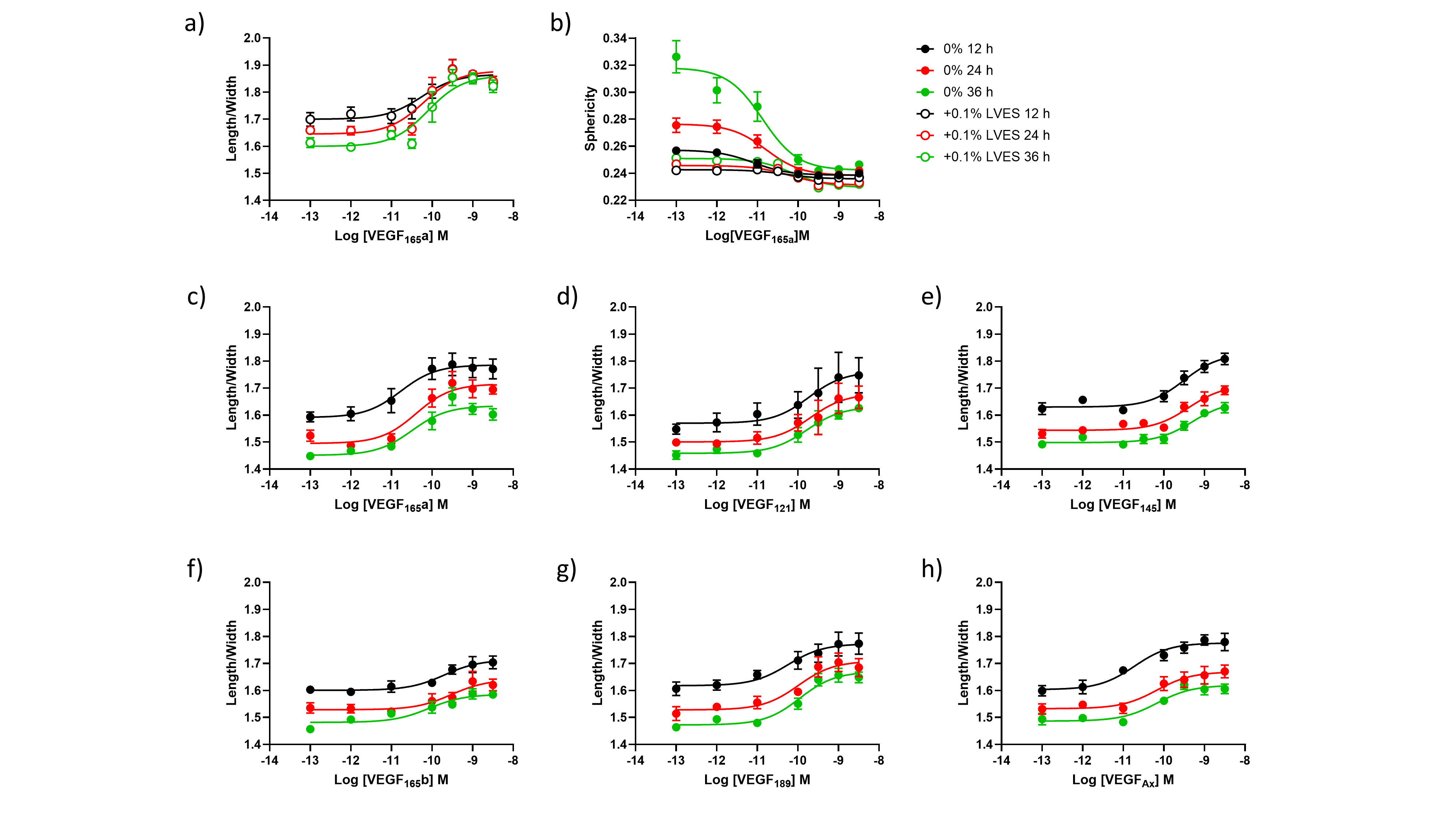

Supplement: Supplementary file 1 — Figure S1. Concentration response curves of mean length to width ratio (a, c–h) or mean sphericity (b) derived from ptychography images at 12 h (black), 24 h (red) and 36 h (green) following treatment with (a) VEGF165a in 0.1% LVES (open circles), (b) VEGF165a in 0.1% LVES (open circles) or vehicle (0% LVES; closed circles), (c) VEGF165a in vehicle (0% LVES; closed circles), (d) VEGF121a in vehicle, (e) VEGF145a in vehicle, (f) VEGF165b in vehicle, (g) VEGF189a in vehicle or (h) VEGF165Ax in vehicle. Data are mean ± SEM of 3–7 separate experiments (see Table 2). [file JMI-300-234-s001.TIF]
